# Hippocampography Guides Consistent Mesial Resections in Neocortical Temporal Lobe Epilepsy

**DOI:** 10.1155/2016/3581358

**Published:** 2016-09-14

**Authors:** Marcus C. Ng, Ronan Kilbride, Mirela Simon, Emad Eskandar, Andrew J. Cole

**Affiliations:** ^1^Section of Neurology, Department of Internal Medicine, Health Sciences Centre, University of Manitoba, Winnipeg, MB, Canada; ^2^Clinical Neurophysiology, Beaumont Hospital, Dublin, Ireland; ^3^Epilepsy Service, Department of Neurology, Massachusetts General Hospital, Harvard Medical School, Boston, MA, USA; ^4^Department of Neurosurgery, Massachusetts General Hospital, Harvard Medical School, Boston, MA, USA

## Abstract

*Background.* The optimal surgery in lesional neocortical temporal lobe epilepsy is unknown. Hippocampal electrocorticography maximizes seizure freedom by identifying normal-appearing epileptogenic tissue for resection and minimizes neuropsychological deficit by limiting resection to demonstrably epileptogenic tissue. We examined whether standardized hippocampal electrocorticography (hippocampography) guides resection for more consistent hippocampectomy than unguided resection in conventional electrocorticography focused on the lesion.* Methods*. Retrospective chart reviews any kind of electrocorticography (including hippocampography) as part of combined lesionectomy, anterolateral temporal lobectomy, and hippocampectomy over 8 years . Patients were divided into mesial (i.e., hippocampography) and lateral electrocorticography groups. Primary outcome was deviation from mean hippocampectomy length.* Results.* Of 26 patients, fourteen underwent hippocampography-guided mesial temporal resection. Hippocampography was associated with 2.6 times more consistent resection. The range of hippocampal resection was 0.7 cm in the mesial group and 1.8 cm in the lateral group (*p* = 0.01). 86% of mesial group versus 42% of lateral group patients achieved seizure freedom (*p* = 0.02).* Conclusions*. By rationally tailoring excision to demonstrably epileptogenic tissue, hippocampography significantly reduces resection variability for more consistent hippocampectomy than unguided resection in conventional electrocorticography. More consistent hippocampal resection may avoid overresection, which poses greater neuropsychological risk, and underresection, which jeopardizes postoperative seizure freedom.

## 1. Introduction

**Figure 1:**
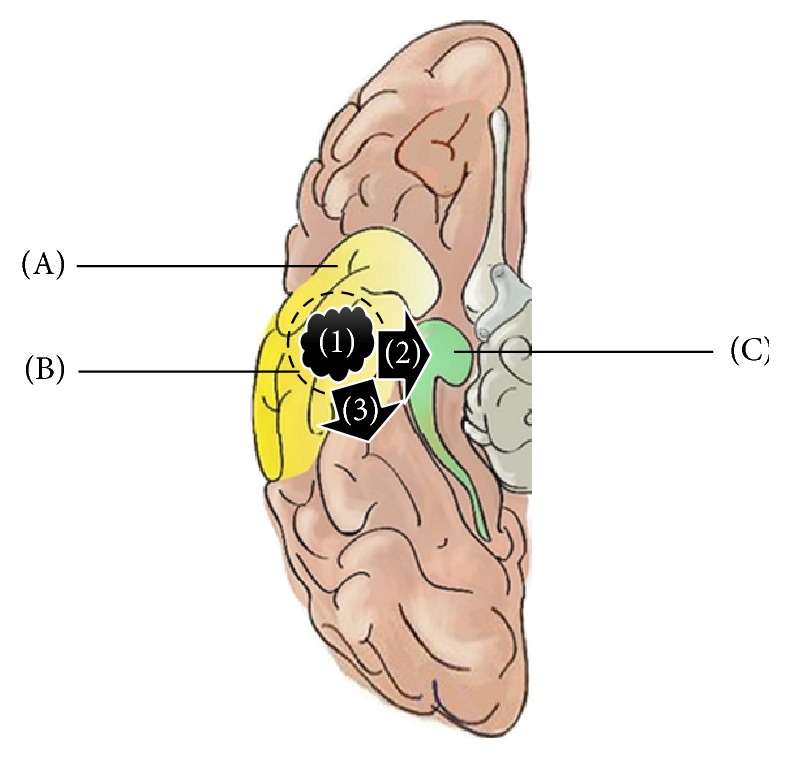
Options in lesional neocortical temporal lobe epilepsy (NTLE) surgery. (A) Anterolateral temporal lobectomy (ALTL) with sparing of mesial temporal structures. (B) Total lesionectomy. (C) Hippocampectomy. (1) Lesion solely confined to temporal neocortex; (2) lesion in temporal neocortex with extension into the adjacent mesial temporal region; (3) lesion in temporal neocortex with extension into the adjacent extratemporal region.

Most neocortical temporal lobe epilepsy (NTLE) is lesional [1]. When refractory to medications, surgery is pursued but the extent of resection is controversial [2]. The most conservative option is isolated lesionectomy. Another option is lesionectomy with anterolateral temporal lobectomy (ALTL) while avoiding mesial temporal structures. A more aggressive option combines lesionectomy, ALTL, and hippocampectomy (Figure 1). However, even when the neocortical lesion appears to spare mesial structures, hippocampal abnormalities have been demonstrated on magnetic resonance spectroscopy and electrocorticography (ECoG) [3, 4]. While diseased neocortex may be justifiably resected to maximize the chance of postoperative seizure freedom, extending resection into hippocampus is controversial due to the greater risk of neuropsychological deficit [5, 6]. Intraoperative ECoG has been used to strike a balance between seizure freedom and neuropsychological risk in order to clarify optimal resection extent [7, 8]. ECoG can maximize the likelihood of seizure freedom by identifying normal-appearing epileptogenic tissue for resection. ECoG can also minimize the chance of neuropsychological deficit by limiting resection to only demonstrably epileptogenic tissue. However, most ECoG studies do not specifically pertain to NTLE and they adopt heterogeneous methods [9, 10]. Even when ECoG has been used in NTLE, ECoG has conventionally focused on the lateral neocortical lesion rather than mesial temporal structures [9, 10]. When hippocampectomy is performed in these cases, mesial temporal resection has not necessarily been guided by ECoG [11, 12]. Unguided hippocampectomy risks yielding widely variable and arbitrary mesial temporal resections that either miss normal-appearing epileptogenic hippocampal tissue to jeopardize postoperative seizure freedom, or risks unnecessarily overextending hippocampal resection to yield greater neuropsychological deficit. In this study, we assessed the specific impact of standardized mesial temporal ECoG on hippocampectomy in NTLE (in addition to lesionectomy and ALTL). We hypothesize that standardized intraoperative hippocampal ECoG (“hippocampography” or “EHG”) guides mesial temporal resection to yield more consistent and less variable hippocampectomy than unguided mesial temporal resection in “conventional lateral ECoG.”

## 2. Methods

**Figure 2:**
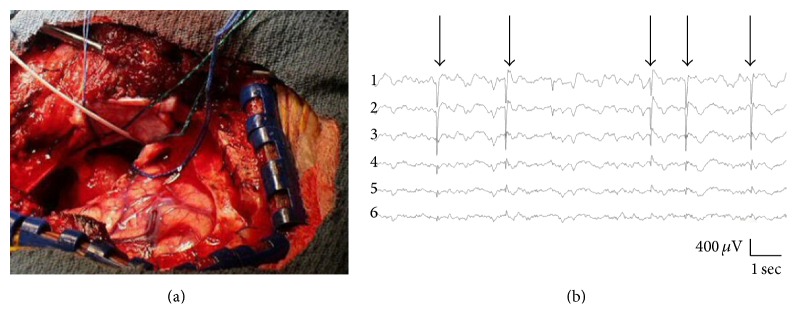
Intraoperative hippocampal electrocorticography (hippocampography). (a) Hippocampography technique. Immediately following resection of the temporal pole prior to possible hippocampectomy, a depth electrode (white wire) placed in an anteroposterior orientation runs parallel to the mesial wall of the inferior horn of the lateral ventricle. The electrode contains 4–8 recording sites with distal contacts located posteriorly by the hippocampus. (b) Hippocampography EEG. Epileptiform discharges (arrows on spike-wave discharges) on referential montage. Contact #1 is posterior and contact #6 is anterior.

We obtained approval from the Massachusetts General Hospital Institutional Review Board to perform retrospective chart reviews on lesional NTLE patients from 2006 to 2014 who underwent any kind of ECoG (including EHG) during lesionectomy, ALTL, and hippocampectomy. Epileptologists diagnosed NTLE based on semiology, surface and intracranial EEG, 3- or 7-Tesla MRI, interictal PET, MRS, MEG, and ictal SPECT. Lesional NTLE required an MRI-visible lesion in temporal neocortex with epileptogenic substrate on histopathology. Patients had undergone “mesial ECoG” or conventional “lateral ECoG.” The mesial ECoG group was comprised of EHG in 14 patients. In tandem with ALTL, a depth or strip electrode ranging from 6 cm to 8 cm was placed in or along the ventricular hippocampal surface (Figure 2(a)). A neurophysiologist reviewed live EHG in referential and bipolar montages (Figure 2(b)). Baseline activity was observed under inhalational anesthesia for at least 5 minutes and after intravenous alfentanyl provocation (30 *μ*g/kg up to 2 doses) with particular attention 90–120 seconds after bolus for peak dose effect. Mesial ECoG was augmented by foramen ovale electrodes in 3 patients. The lateral ECoG group was comprised of neocortical subdural grid or strip electrodes (ranging from 8 to 64 contacts) over the lateral temporal, ipsilateral central, parietal, and/or frontal regions in 12 patients to identify extrahippocampal epileptogenic tissue for resection, or eloquent juxtalesional tissue as the boundary of resection. Mesial resection in lateral ECoG was based on visual inspection, except for one patient who underwent both mesial and lateral ECoG. Choice of ECoG was based on whether the neocortical lesion was close to, or extended into, adjacent mesial temporal (“NTLE+M”) or extratemporal regions (“NTLE+E”) on MRI. Where the lesion extended into both regions (“NTLE+M+E”), lateral ECoG was preferred. EHG was also used in comorbid “dual pathology” mesial temporal sclerosis (MTS). The primary study outcome was deviation from mean hippocampectomy length in each group. Secondary outcomes were absolute hippocampectomy length, seizure freedom, and any worsening or improvement of neuropsychological performance. Seizure freedom was defined as Engel Class I [13]. Engel Class was determined from the last available clinical note. We specified hippocampectomy length, age, gender, lateral ECoG, lesional spread outside temporal neocortex, comorbid MTS, and follow-up duration as possible confounders of seizure freedom. Any postoperative changes in neuropsychological performance were based on data one year after surgery. Both univariate statistical analysis (*t*-test and chi-squared test) and stepwise forward and backward Wald test multivariate logistic regression used SPSS software.

## 3. Results

**Figure 3:**
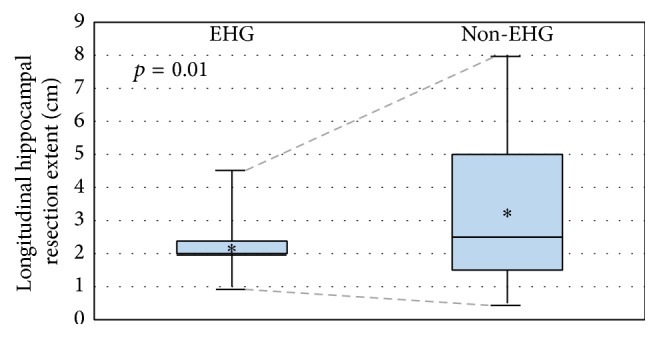
Hippocampal resection variability. Comparison of interquartile ranges of longitudinal hippocampal resection extents in patients undergoing hippocampography (EHG) versus other electrocorticography (non-EHG). Asterisks (*∗*) denote mean longitudinal hippocampal resection extent per group. Statistical significance (*p* = 0.01) refers to the difference in hippocampectomy length variability (range of the deviation from the mean) between groups (dotted lines).

Out of 26 lesional NTLE patients who underwent lesionectomy, ALTL, and hippocampectomy, 14 patients in the mesial ECoG group underwent EHG-guided mesial resection while 12 patients underwent unguided mesial resection in the lateral ECoG group. There were four MRI-occult dual pathology MTS cases, which were equally distributed (two each) among the mesial and lateral ECoG groups. Six surgeries occurred in the nondominant hemisphere (Table 1). Mean absolute hippocampectomy length was 2.2 cm in the mesial ECoG group and 3.1 cm in the lateral ECoG group (*p* = 0.20, Table 1 and Figure 3). The range of deviation from mean hippocampectomy length was 0.7 cm (0.2 cm–2.3 cm) in the mesial ECoG group and 1.8 cm (0.6 cm–4.9 cm) in the lateral ECoG group (*p* = 0.01, Table 1 and Figure 3). Regarding secondary outcomes, more patients achieved seizure freedom in the mesial than lateral ECoG group on univariate analysis (86% versus 42%, *p* = 0.02, Table 2). On univariate analysis of possible confounders of seizure freedom, NTLE+M (lesional invasion of mesial temporal structures) and NTLE+M+E (simultaneous mesial temporal and extratemporal lesional invasion) were additional factors which were also significantly associated with achieving postoperative Engel Class I at time of last follow-up (Table 2). In a forward selection multivariate logistic regression model, the only significant factor was NTLE+M+E, which was negatively associated with seizure freedom (model *p* = 0.015). Using a backward selection model, the only significant factor was NTLE+M, which was positively associated with seizure freedom (model *p* = 0.005). EHG was not significantly associated with seizure freedom in either multivariate model. Only 6 of 26 patients (2 mesial ECoG and 4 lateral ECoG patients) had longitudinal preoperative and postoperative neuropsychological data (Table 3). While most patients underwent preoperative testing, many did not attend postoperative testing or they were lost to follow-up. Total available longitudinal neuropsychological data were insufficient for statistical analysis.

## 4. Discussion

Hippocampography (EHG) in lesional NTLE patients guided mesial temporal resection to yield 2.6 times more consistent hippocampectomy than unguided mesial temporal resections in patients who did not undergo EHG. This finding is consistent with our hypothesis that EHG reduces the variability in mesial temporal resection which is inherent with an unguided arbitrary approach. Although hippocampectomy variability was reduced by EHG, there was no significant difference in overall absolute hippocampectomy length between mesial and lateral ECoG groups. This may relate to very conservative and very expansive hippocampectomy outliers in the lateral ECoG group which balanced one another to settle at a mean which was not significantly different from the mesial ECoG group. Reducing hippocampectomy variability, however, to arrive within a tight optimal resection range is critical in balancing the competing goals of maximizing seizure freedom and minimizing postoperative neuropsychological risk. Unnecessarily conservative hippocampal resections risk missing excision of normal-appearing epileptogenic tissue. Unnecessarily expansive hippocampal resections risk greater postoperative neuropsychological deficit. Our study also assessed postoperative seizure freedom and neuropsychological changes as secondary outcomes. On univariate analysis, EHG was associated with greater postoperative seizure freedom. This finding may relate to EHG detection of unremarkable epileptogenic hippocampal tissue otherwise missed on visual inspection. Because most EHG was conducted using depth electrodes, it is also possible that EHG may have inadvertently destroyed epileptogenic hippocampal tissue while sampling it. Unfortunately, we lacked sufficient data to comment on postoperative neuropsychological outcomes. Based on our findings, we conclude that if the decision has been made to include hippocampal resection as part of NTLE surgery, then EHG is a readily applied intraoperative ECoG technique which guides mesial temporal resection to yield the benefits of more consistent hippocampal resection.

These findings are subject to many limitations. First, there is no consensus on a “gold standard” surgery for lesional NTLE. In this study, the standard was pegged to our conventional practice of lateral ECoG using neocortical subdural grid and strip electrodes. Lateral ECoG patients formed the “control” unguided hippocampectomy group against which the “test” EHG-guided hippocampectomy group was compared. Although lateral ECoG may not be “gold standard,” our results, however, are applicable to any institution-specific “conventional” ECoG practice which does not guide mesial temporal resection. Another limitation of this study is follow-up duration. Although this factor was not found to be a significant confounder of postoperative seizure freedom in either univariate or multivariate analysis, the follow-up duration for Engel Class I patients was less than one year in 7 of 17 patients, which was equally distributed between the mesial and lateral ECoG groups. This suggests caution in the interpretation of seizure freedom durability in either ECoG group. Limitations also apply to study outcomes. Regarding hippocampectomy length (a one-dimensional measurement), it is possible that there may have been a relationship between hippocampectomy volume (a three-dimensional construct) and EHG. Unfortunately, volumetric data were not available from surgical reports and we were unable to perform detailed volumetry on postoperative MRI of lesionectomy and ALTL. Regarding the secondary outcome of postoperative seizure freedom, NTLE patients who underwent EHG may have “converted” to the better prognosis of MTLE as a result of selection bias which favored EHG where the temporal neocortical lesion was close to the mesial temporal region, invaded into the mesial temporal region or, there was dual pathology MTS (“NTLE+M”). On the other hand, lateral ECoG without EHG was favored when the lesion was close to, or extended into, the adjacent extratemporal region (“NTLE+E”). These lateral ECoG cases may have “converted” to the worse prognosis of extratemporal epilepsy (NTLE+E). However, some recent studies did not find a difference in seizure freedom between MTLE and NTLE [1, 14]. The historical difference in outcome has been attributed to poor patient selection, poor localization, and incomplete neocortical lesionectomy [15–20]. Regarding the secondary outcome of postoperative neuropsychological outcome, we did not have adequate test data to perform formal statistical analysis. While most patients experienced postoperative declines, especially on the Boston Naming Test, there were select patients who achieved better scores in visual memory and auditory logical memory. Nevertheless, there were insufficient data from which to draw strong conclusions on postoperative neuropsychological outcomes.

While we found that EHG tailored more consistent mesial temporal resections in lesional NTLE to significantly reduce hippocampectomy variability when compared to surgeries using ECoG with unguided hippocampectomy, we were unable to directly correlate reduced resection variability with either secondary outcome of improved seizure freedom and/or less neuropsychological deficit. Further elucidating the impact of consistent EHG-guided resections on seizure freedom and neuropsychological outcome depends on larger numbers of patients in a future randomized controlled trial from different institutions with extended follow-up periods and equal numbers of patients undergoing surgeries in the dominant and nondominant hemispheres. These NTLE patients should have more uniform and “pure” lesion locations confined to the temporal neocortex, as verified by dedicated volumetry, which can also precisely delineate anatomical boundaries and resection extents. All patients should have preoperative and postoperative neuropsychological testing at standardized times with standardized neuropsychological metrics. We hope that the findings from our study help inspire such a trial in order to rigorously assess the important yet unanswered issue of what constitutes the “gold standard” surgery in NTLE. Data from a future trial may also help elucidate the unique potential of EHG in striking an ideal balance that maximizes seizure freedom and minimizes neuropsychological deficit in dominant hemispheric cases of lesional NTLE.

## Figures and Tables

**Table 1:** Patient characteristics.

Pt	Mesial ECoG (EHG-guided hippocampectomy)	Lateral ECoG (unguided hippocampectomy)	Side	Age (y)	Sex	Lesion histopathology	Lesion location on MRI	MTS	Hippocampectomy (cm)	Follow- up (y)	Engel I
1	EHG(D)		D^H^	38	M	Glioma	NTLE+M	No	1	1.15	Yes
2	EHG(S)		D^H^	15	F	Ganglioglioma	NTLE+M+E	No	1	4.05	No
3	EHG(S)		ND^H^	24	F	Encephalomalacia	NTLE	No	1.5	0.8	Yes
4	EHG(D)		D^H^	24	M	Dysplasia	NTLE+M	Yes	2	2.05	Yes
5	EHG(D)+FO		D^H^	20	M	Encephalocele	NTLE	Yes^MRI-^	2	0.79	Yes
6	EHG(D)		D^H^	27	M	Dysplasia	NTLE	Yes^MRI-^	2	1.8	Yes
7	EHG(D)	Grid	ND^H^	7	M	Encephalomalacia	NTLE+M+E	Yes	2	0.03	Yes
8	EHG(D)		D^H^	30	F	Dysplasia	NTLE	No	2	0.11	Yes
9	EHG(D)		D^H^	66	M	Oligodendroglioma	NTLE+M	No	2	1.3	Yes
10	EHG(D)+FO		D^H^	33	M	Glioma	NTLE+M	No	2	4.22	Yes
11	EHG(D)+FO		D^H^	33	M	Glioma	NTLE+M	Yes	2.5	2.5	Yes
12	EHG(D+S)		ND^HW^	31	F	Astrocytoma	NTLE+M	No	2.5	2.79	Yes
13	EHG(D)		ND^H^	51	F	Dysplasia	NTLE	No	4	1.25	No
14	EHG(D)		ND^H^	46	F	Glioma	NTLE+M	Yes	4.5	0.33	Yes
15		Grid	D^HW^	18	F	Dysplasia	NTLE	No	0.5	4.63	Yes
16		Strip	D^H^	44	M	Glioma	NTLE+E	Yes	1.5	0.26	Yes
17		Grid + strip	D^H^	27	M	Dysplasia	NTLE+E	Yes	1.5	1.1	No
18		Grid	D^H^	54	F	Oligodendroglioma	NTLE+M+E	No	1.5	2.01	No
19		Grid	D^HW^	34	M	eMultipl^1^	NTLE+M+E	No	2	4.2	No
20		Grid	D^H^	29	F	Glioma	NTLE	Yes^MRI-^	2.5	2	No
21		Grid	D^H^	62	M	Encephalomalacia	NTLE	Yes^MRI-^	2.5	2.23	No
22		Grid	D^H^	24	M	Astrocytosis	NTLE+M+E	No	2.5	2.6	No
23		Strip	D^H^	22	F	Dysplasia	NTLE+M+E	Yes	5	2.16	Yes
24		Grid	D^H^	74	F	Encephalomalacia	NTLE+E	No	5	0.03	Yes
25		Grid + strip	D^H^	33	F	Dysplasia	NTLE+M+E	No	5	3.1	No
26		Grid + strip	D^H^	32	M	Multiple^2^	NTLE+M+E	Yes	8	1.07	Yes

^1^Dysplasia, Heterotopia, and Polymicrogyria; ^2^Encephalomalacia and Polymicrogyria; EHG(D): hippocampography using depth electrode; EHG(S): hippocampography using strip electrode; FO: foramen ovale electrode; D: dominant hemisphere; ND: nondominant hemisphere; H: hemispheric dominance based on handedness; W: hemispheric dominance based on Wada test; NTLE+M: neocortical temporal lobe epileptogenic lesion with mesial temporal invasion; NTLE+E: neocortical temporal lobe epileptogenic lesion with extratemporal invasion; NTLE+M+E: neocortical temporal lobe epileptogenic lesion with mesial temporal and extratemporal invasion.

**Table 2:** Group characteristics.

	Mesial ECoG (EHG-guided hippocampectomy)	Lateral ECoG (unguided hippocampectomy)	Group differences *p *(univariate)	Association with Engel I *p *(univariate)
*Primary outcome*				
Hippocampectomy variability (deviation from mean, cm)	0.7 (0.2–2.3)	1.8 (0.6–4.9)	0.01^*∗*^	n/a
*Secondary outcomes*				
Absolute hippocampectomy length (cm)	2.2 (1–4.5)	3.1 (0.5–8)	0.20	0.74
Engel Class I	12 (86%)	5 (42%)	0.02^*∗*^	n/a
*Possible confounders of secondary outcome Engel Class I*				
Age	31.8 (7–66)	37.8 (18–74)	0.36	0.64
Male gender	8 (57%)	6 (50%)	0.72	0.48
Comorbid MTS	6 (43%)	6 (50%)	0.72	0.34
NTLE+M^*∗∗*^	7 (50%)	1 (8%)	0.051	0.02^*∗*^
NTLE+E	1 (7%)	3 (25%)	0.07	0.96
NTLE+M+E^*∗∗*^	1 (7%)	5 (42%)	0.002^*∗*^	0.046^*∗*^
Follow-up duration	1.7 (0.03–4.22)	2.1 (0.03–4.63)	0.40	0.07

^*∗*^Statistically significant on univariate analysis.

^*∗∗*^Statistically significant on multivariate analysis: NTLE+M was positively associated with Engel I; NTLE+M+E was negatively associated against Engel I.

NTLE+M: neocortical temporal lobe epileptogenic lesion with mesial temporal invasion; NTLE+E: neocortical temporal lobe epileptogenic lesion with extratemporal invasion; NTLE+M+E: neocortical temporal lobe epileptogenic lesion with mesial temporal and extratemporal invasion; n/a: not applicable.

**Table 3:** Postoperative neuropsychological testing absolute percentile changes.

Pt	ECoG	Side	Hippocampectomy (cm)	WAIS3			WMS3					BNT	
				FSIQ	VIQ	PIQ	Visual memory	Spatial memory	Remind test	Auditory verbal processing	Auditory logical memory	Naming	Auditory naming
10	Mesial EHG	D	2		−9	−3	0	0	−48	0	−62	−22.5	
11	Mesial EHG	D	2.5					−42	1				
16	Lateral Strip	D	1.5		−4		75	0		−1	20	5	
19	Lateral Grid	D	2	−2	−10	7	−59	0	−20	25	41	0	−2
20	Lateral Grid	D	2		3	−3	25	−72	−17	0	0		−71
21	Lateral Grid	D	2.5		−1	12	25		−1	0	66	0	−2

ECoG: electrocorticography; EHG: hippocampography; D: dominant hemisphere; WAIS3: Wechsler adult intelligence scale 3rd edition; FSIQ: full scale intelligence quotient; VIQ: verbal intelligence quotient; PIQ: performance intelligence quotient; WMS3: Wechsler memory scale 3rd edition; BNT: Boston Naming Test.
